# Threshold Resistive Switching in Inorganic Lead-Free Cesium–Bismuth Iodide Perovskite for Neuron Emulation

**DOI:** 10.1021/acsaelm.5c00516

**Published:** 2025-04-04

**Authors:** Michalis Loizos, Konstantinos Chatzimanolis, Katerina Anagnostou, Konstantinos Rogdakis, Emmanuel Kymakis

**Affiliations:** † Department of Electrical & Computer Engineering, Hellenic Mediterranean University (HMU), Heraklion 71410, Crete Greece; ‡ Institute of Emerging Technologies, University Research and Innovation Center (HMU), Heraklion 71410, Crete Greece

**Keywords:** threshold switching memristor, neuromorphic computing, lead-free perovskites, neurons, volatile memristor, cesium−bismuth perovskite

## Abstract

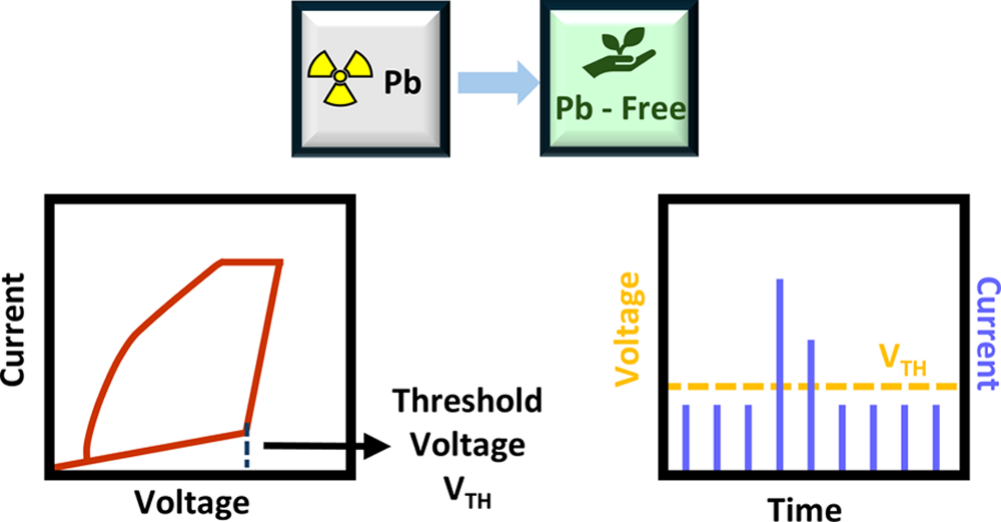

High-performance halide-based perovskite memory devices have been developed, exhibiting a variety of synaptic and neuronal functions based on nonvolatile and volatile or threshold switching memristors, respectively, compatible with low power consumption. However, the key ingredient in these perovskite-based systems is the presence of highly toxic lead, which hinders their further development and commercial use. A lead-free perovskite approach for memristive applications could enable sustainable devices, opening the path for practical applications. Herein, we report on the fabrication and characterization of a threshold resistive switching device using solution-based manufacturing, based on a lead-free, all-inorganic perovskite, namely cesium–bismuth iodide (Cs_3_Bi_2_I_9_) perovskite. The memristive device exhibits threshold switching current–voltage (I–V) characteristics with an ON/OFF ratio of >10^4^, while operating in the 0 V–5 V range and exhibiting a cycling endurance of 650 cycles with reproducible behavior. Furthermore, linear long-term, threshold-dependent potentiation protocols, accompanied by abrupt resistance suppression under depression protocols, are demonstrated. The volatile nature of memristive switching allowed the implementation of current spiking activation, similar to neuron spiking protocols, thus opening the path for neuronal emulation. These results can further advance the development of environmentally friendly perovskite memory systems for neuromorphic computing applications, providing a cost-effective alternative to oxide-based devices.

## Introduction

The current central technological application of metal halide perovskites is their integration into high-performing, solution-processed solar cells and modules. Ongoing research efforts are continuously pushing upward the efficiency of metal-halide perovskite solar cells (PSCs) with impressive achievements, such as over 26% efficiency,^1−3^ while commercialization attempts focus on implementing roll-to-roll methods for large-scale production of solar modules.^4,5^ Nonetheless, apart from the limited stability, the use of highly toxic lead (Pb) is a major environmental concern that hinders the commercial use of PSCs. To mitigate the environmental impact of lead, lead-free perovskite compounds have been implemented as well,^6^ to replace the B-site cation in the general ABX_3_ formula, where A is a monovalent cation, B is a divalent metal cation, and X is a halogen, typically I or Br. Potential replacements for the B-site cation, which is commonly Pb, include compounds based on Bi, Sn, Ge, and Sb.

The mixed conduction type of perovskite-based materials, involving both ionic and electronic charge flow, which is often tunable by electrical and optical stimulation, is the origin of their emerging properties with advantageous applications in various fields of electronics. Specifically, devices based on hybrid organic/inorganic mixed halide perovskites have demonstrated superior performance across various optoelectronic applications, such as solar cells, photodetectors, memristive devices, and radiation sensors.^7,8^ A field with strong scientific and technological interest today is the development of a novel computational paradigm based on resistive switching (RS) memories, which go beyond von Neumann computation architectures. The observed hysteresis in perovskite-based devices, associated with ion migration^9^ that has a low energy onset, renders them suitable for RS memory applications. In addition, perovskite-based RS devices and crossbars^10^ have been developed for implementing artificial synapses and neuron emulation.^11−15^ Moreover, the excellent optoelectronic response of perovskites enables the implementation of light-tunable memory devices and synapses.^16−19^

High-performance perovskite RS devices have been developed, demonstrating high ON/OFF ratios of up to 10^9^,^20^ with switching speeds on the order of ns,^21^ good cycling endurance,^22,23^ and state retention.^24^ The solution processing of perovskites enables cost-effective RS device fabrication on flexible substrates,^25,26^ while efforts focus on more sustainable fabrication routes that could further reduce manufacturing costs.^27,28^ The typical structure of a perovskite memristive device reported in the literature is that of a simple metal–insulator–metal configuration; however, other interfacial materials and configurations have been exploited to maximize the switching effect of the devices. Notably, an approach using a solar cell structure exhibiting memristive behavior has been reported, which can operate as a dual-mode device performing either energy harvesting or synaptic functions. These devices, termed memristive PSCs, could pave the way toward self-powered IoT edge computing;^29^ however, their Pb content could pose an issue regarding sustainability. Experimental parameters, such as the active layer and the interfacial layers used, can greatly affect the overall device performance.^30^ In this regard, high-performance lead-free perovskite compounds^31^ have been explored as an alternative to replace Pb in RS memories.^32^ Among the various elements that have been tested, Bi has gained significant attention along with Sn, while other options such as Sb and Cu have also been considered.^33−35^ Apart from the interfaces, tuning the thickness of the perovskite switching layer can lead to different types of filaments as the dominant mechanism, revealing an interplay between vacancy-based and metal-based filaments.^36^ The surface morphology of the perovskite film is also vital for achieving reliable switching. Lee et al. have shown that controlling the grain size of the perovskite through the fabrication process can affect the switching properties. Specifically, increasing the grain size led to a decrease in the HRS current, hence an increase in the ON/OFF ratio.^37^

Bismuth-based compounds are known to form A_3_Bi_2_X_9_ chemical structures, where the A-site cation can be occupied by Methylammonium (MA), Formamidinium (FA), Cesium (Cs), or Rubidium (Rb). The B-site cation is either Bi or Sb, and the X site is either I, Br, or Cl. Typically, the crystal structure of these systems belongs to the P63/mmc space group with a hexagonal structure. These perovskites can form a 0D structure through a close packing of [Bi_2_I_9_]^3–^ dimer units; hence, this phase is often referred to as a dimer. Alternatively, a 2D layered crystal structure can also form, for instance, Cs_3_BiI1_10_.^38^ These compounds, however, are not ideal for optoelectronic applications, such as solar cells or optoelectronic memristors, due to their wide, indirect bandgaps (2–3 eV) and high exciton binding energy.^39^ Furthermore, these structures contain many defects that can act as recombination centers, further limiting the resulting efficiency and charge mobility, which is the main reason why most of these PSCs exhibit efficiencies below 1%.^40,41^

Contrary to the case of solar cells, A_3_Bi_2_X_9_ perovskites have the potential for RS memory applications, as Bi is a relatively stable and nontoxic alternative compared to Pb, while maintaining the ability to form conductive filaments due to active metal cations or iodine migration, which possesses low activation energy. Remarkably, high-performance, Pb-free RS devices with a high ON/OFF ratio and low electric field operation have been developed for the case of Cs_3_Bi_2_I_9_,^42,43^ demonstrating synaptic functionality emulation.^44−46^ While nonvolatile memristors can maintain their resistance states over time, volatile systems, on the other hand, possess transient dynamics with a spontaneous transition to the high resistance state (HRS) through the ion diffusion process, and therefore threshold switching memristors cannot store information. The diffusive dynamics^47^ of these systems allow them to mimic neuronal activity based on biorealistic neuron modeling, thus enabling applications that evolve spiking neural networks (SNNs).^48,49^ There are a limited number of studies regarding lead-free RS systems with volatile behavior. Zawal et al. developed a diffusive lead-free system based on *n*-butylammonium bismuth iodide (BABI) perovskite. The device operated through transient conductive filaments induced by iodine vacancies. A firing response in agreement with the leaky-integrate-and-fire (LiF) neuron model was realized. Finally, the experimental data were used for the classification task of pattern recognition.^50^

In this study, we demonstrate the fabrication of a lead-free, sustainable inorganic perovskite memory device based on Cs_3_Bi_2_I_9_ using solution-based processing, developed in an inverted solar cell structure. A volatile memristive system with threshold switching I–V characteristics is realized with an ON/OFF ratio exceeding 10^4^, while operating at relatively low electric fields, which also exhibits a cycling endurance of 650 cycles. Typical devices exhibit low variability after multiple sequential I–V scans and across different batches. Electrical pulsed characterization enabled a long-term, threshold-dependent linear potentiation protocol accompanied by an abrupt depression behavior upon electric field removal, confirming the volatile nature of the device. Finally, pulsed characterization revealed the ability of the device to emulate basic neuronal functions such as spontaneous spiking, showcasing its potential for developing high-performance, cost-effective, and environmentally friendly perovskite memories for neuromorphic computing applications.

## Experimental Section

### Materials and Methods

Dimethylformamide (99.8%, anhydrous), dimethyl sulfoxide (99.9%, anhydrous), bismuth iIodide (99%), chlorobenzene (99.8%, anhydrous), 2,9-dimethyl-4,7-diphenyl-1,10-phenanthroline (bathocuproine, BCP, 96%), and isopropyl alcohol (99.5%, anhydrous) were purchased from Sigma-Aldrich. Poly[bis(4-phenyl)(2,4,6-trimethylphenyl)amine] (poly(triarylamine), PTAA, *M*_w_ = 20–70 kDa) was purchased from Solaris Chem. Cesium iodide (CsI, trace metals basis, 99.999%) was purchased from Alfa Aesar. Toluene (>99.7%, anhydrous) was purchased from Honeywell. [6,6]-Phenyl C_60_ butyric acid methyl ester (PC_60_BM, 99%) was purchased from Lumtech.

### Device Fabrication and Characterization

Glass/ITO substrates (Naranjo) were cleaned in an ultrasonic bath via sonication in deionized water, acetone, and isopropyl alcohol for 10 min and were dried under continuous nitrogen flow, followed by oxygen plasma treatment for 5 min. Device fabrication was based on the literature procedure proposed by Khadka et al.,^61^ wherein PTAA (5 mg mL^–1^ in chlorobenzene) was spin-coated at 4000 rpm for 30 s (1000 rpm s^–1^ acceleration) in a N_2_-filled glovebox, followed by annealing at 100 °C for 10 min. The PTAA-coated substrates were then treated again with oxygen plasma for 2 min to improve wettability and were transferred back to the glovebox for further processing. The Cs_3_Bi_2_I_9_ solution with 0.5 M concentration was prepared by mixing CsI and BiI_3_ in a 3:2 ratio in 1 mL DMF:DMSO (7:3), and was heated for 5 h at 70 °C. The perovskite solution was spin-coated at 1500 rpm for 20 s (500 rpm s^–1^ acceleration) and at 3000 rpm for 30 s. Then, 150 μL of chlorobenzene was deposited onto the spinning substrate 20 s after the start of the program. The substrates were then annealed at 120 °C for 1 h. Afterward, PCBM (20 mg mL^–1^ in chlorobenzene) was spin-coated at 800 rpm for 30 s and 4000 rpm for 10 s, followed by annealing at 100 °C for 10 min. BCP (0.5 mg mL^–1^ in 2-propanol) was dynamically spin-coated at 4000 rpm for 45 s. Finally, 100 nm of Ag was thermally evaporated under high vacuum (4 × 10^–6^ mbar). The active area of the memory devices is 4 mm^2^.

The UV–visible (UV–vis) absorption spectrum of the perovskite film was recorded using a Shimadzu UV-2401 spectrophotometer. The X-ray diffraction (XRD) pattern of the perovskite film was recorded with a RIGAKU (Tokyo, Japan) D/MAX-2500 powder diffractometer equipped with monochromated Cu Kα radiation (λ = 1.5418 Å). Atomic Force Microscopy (AFM) images of the perovskite film, with dimensions of 2 μm × 2 μm, were measured using an XE7 microscope (Park Systems). Finally, the steady-state photoluminescence spectrum of the perovskite film was recorded using an FS5 fluorescence spectrophotometer from Edinburgh Instruments. A 478.4 nm laser was used as the excitation source of the excitation.

The current–voltage and pulsed characteristics of the memory device were measured on the commercial ARKEO platform from Cicci S.r.I., while spring probes were attached to the device. Voltage was applied at the Ag top electrode, while the ITO bottom electrode was grounded. The steady-state current–voltage measurements were performed from 0 to 5 V with a 25 mV step and a 200 mV s^–1^ scan rate using compliance currents of 10 μA and 100 μA, respectively. For pulsed measurements, a custom module by Cicci Research was used. The module allows the custom design of waveform pulses. All measurements were performed in ambient air under controlled humidity (30–40%) at 25 °C in the dark.

## Results and Discussion

**Figure 1:**
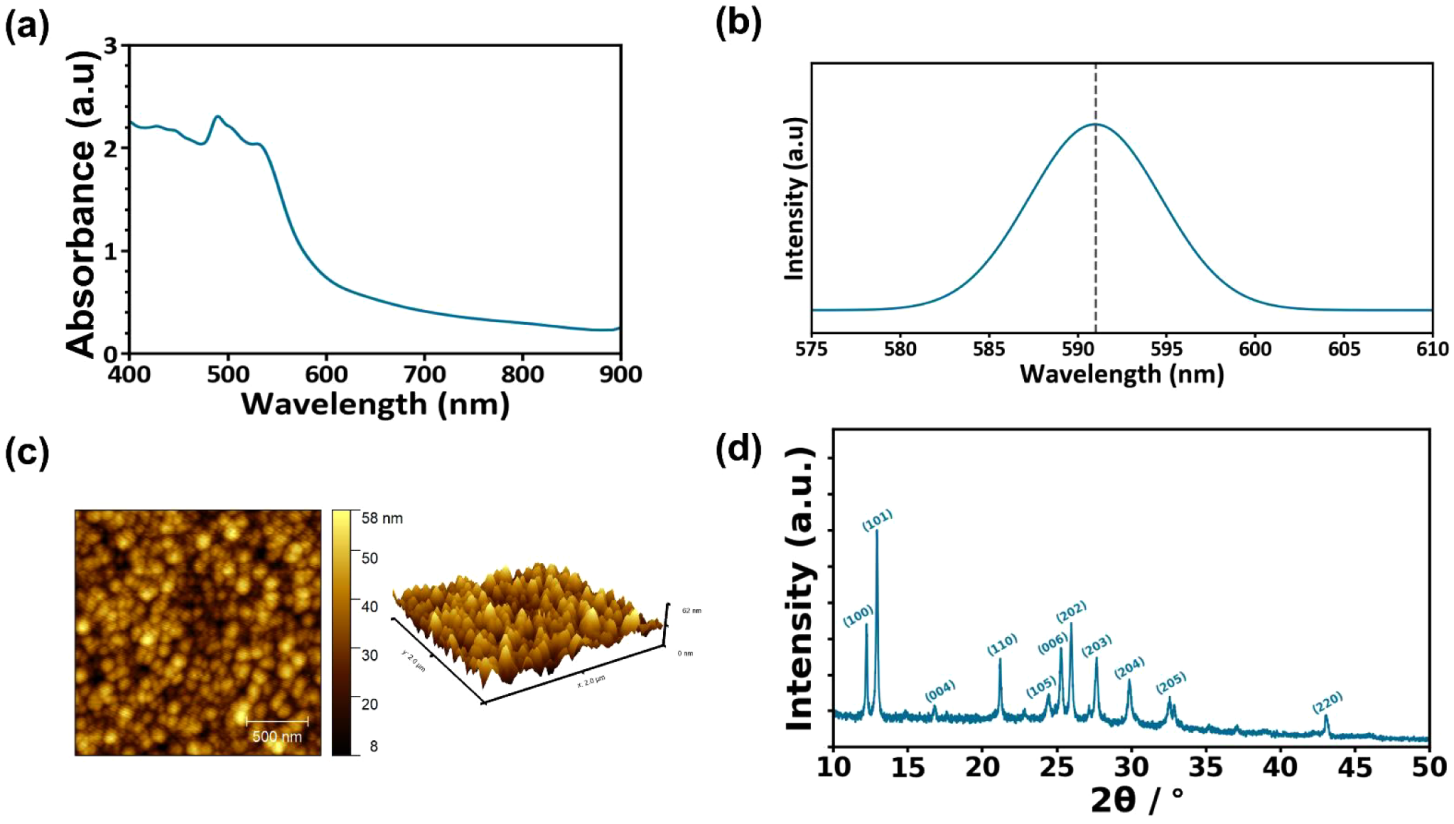
(a) UV–vis spectrum of the Pb-free perovskite film, (b) steady-state photoluminescence spectrum, (c) AFM Image of 2 μm × 2 μm size of the perovskite film on PTAA, and (d) XRD pattern of the Cs_3_Bi_2_I_9_ film.

The thin-film properties of the lead-free, all-inorganic Cs_3_Bi_2_I_9_ (CBI) perovskite were investigated by using UV–vis absorption spectroscopy measurements. The absorption spectrum of Cs_3_Bi_2_I_9_ is depicted in Figure 1a. The characteristic excitonic absorption peak of Cs_3_Bi_2_I_9_ is located at 491 nm.

**Figure 2:**
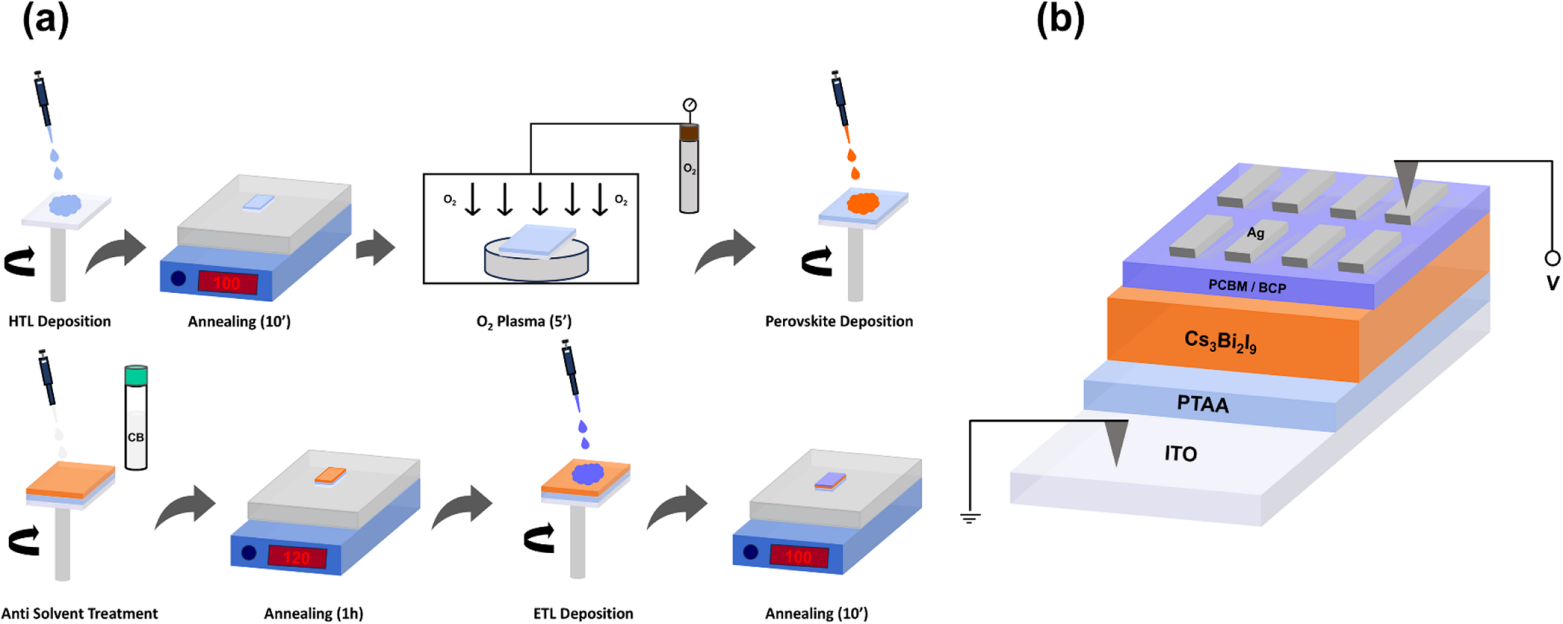
(a) Schematic illustration of procedures followed to complete device fabrication. (b) Final structure of the CBI devices.

The presence of the excitonic absorption peak is indicative of strong quantum confinement due to the 0D nature of the [Bi_2_I_9_]_3_ bioctahedra.^51,52^ The optical, direct bandgap of the obtained CBI film was estimated through the Tauc plot method to be roughly 2.15 eV (Figure S1), which is comparable to the values reported in previous studies^53−55^ as well as calculations from first principles.^56^ Subsequently, the steady-state photoluminescence (PL) spectrum of the CBI film was recorded. As pictured in Figure 1b, the PL emission peak is observed at 591 nm. Atomic Force Microscopy (AFM) images of 2 μm × 2 μm size are shown in Figure 1c and reveal relatively uniformly distributed grains with the presence of a few pinholes (dark regions). The Root Mean Square of the resulting CBI film was estimated at 6.4 nm, indicating a smooth surface. The structural properties of the CBI films were studied by recording the XRD patterns, which are shown in Figure 1d. The XRD pattern revealed peaks located at 2θ values of 12.22°, 12.93°, 16.78°, 21.19°, 24.44°, 25.28°, 25.96°, 27.66°, 29.87°, 32.56°, and 43.04° that correspond to diffractions from the (100), (101), (004), (110), (105), (006), (202), (203), (204), (205), and (220) planes, respectively. These peaks are consistent with the hexagonal CBI perovskite phase with *P*63/*mmc* space group.^57−60^ Furthermore, the thickness of the CBI perovskite film was measured using a profilometer (Figure S2) and is estimated at 900 nm. After evaluating the fundamental properties of the CBI films, we proceeded to fabricate devices to study the resistive switching (RS) characteristics of the CBI-based devices. Device fabrication involved several steps, including PTAA deposition acting as the hole transport layer (HTL), O_2_ plasma treatment to improve the wettability of the films, antisolvent-assisted deposition of the CBI films, PCBM deposition acting as the electron transport layer (ETL), BCP buffer layer deposition, and finally thermal evaporation of the top Ag electrode. All films, except the top electrode, were deposited by spin-coating from the respective solutions. A schematic representation of all steps followed to complete device fabrication is illustrated in Figure 2a, while more details about fabrication can be found in the experimental section. Fabrication of the devices was based on the proposed protocol by Khadka et al.^61^ The structure of the final device is depicted in Figure 2b, with a Glass/ITO/PTAA/CBI/PCBM/BCP/Ag configuration.

**Figure 3:**
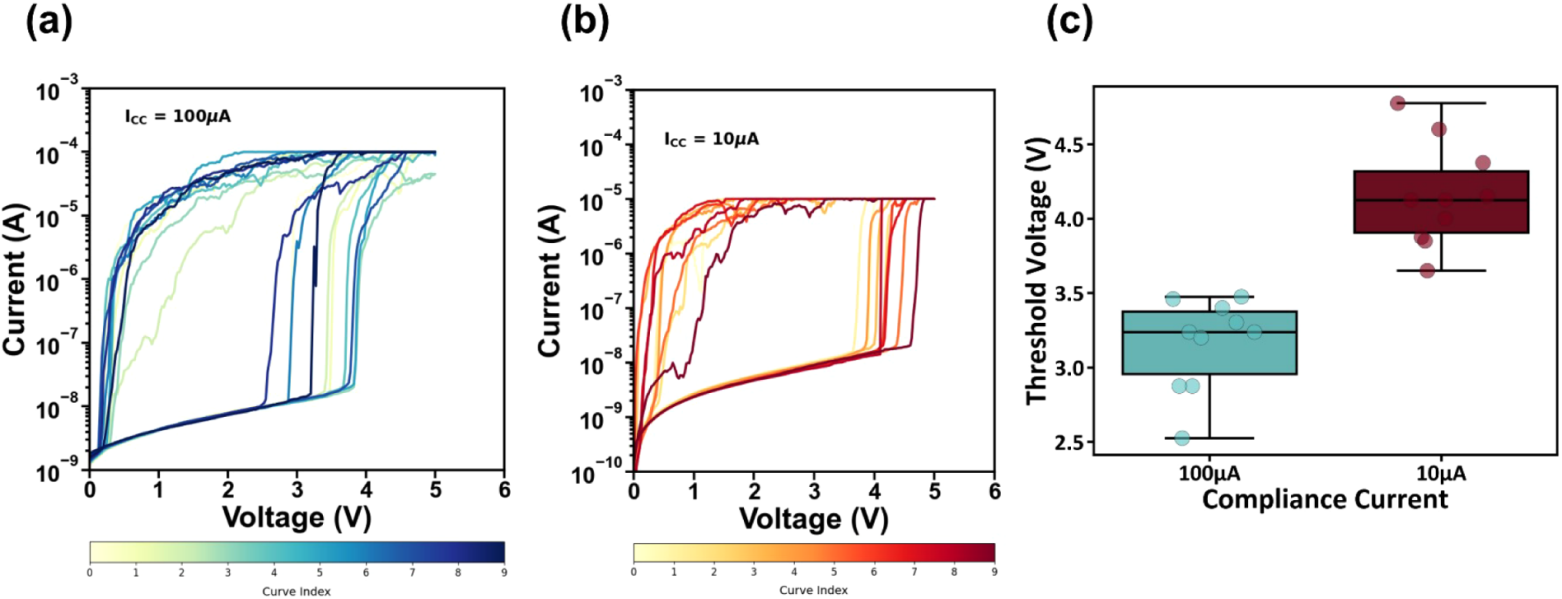
Ten consecutive I–V curves of the CBI volatile memory device with (a) 100 μA and (b) 10 μA compliance current and (c) box plot with threshold voltages obtained for both compliance currents.

The current–voltage characteristics of the resulting CBI Pb-free device were investigated for two cells from the same batch under different compliance current limit values, namely 100 and 10 μA. Figure 3a,b depicts the I–V characteristics of the memory device measured in the sequence 0 V→5 V→0 V for 10 consecutive cycles. In both cases, the system exhibits stable and reproducible RS behavior, starting from a High Resistance State (HRS) with current levels of a few nA up to 10 nA. Subsequently, an abrupt switching occurs in both cases, corresponding to the formation (SET) process. The current amplitude then reaches values on the order of 0.1 mA and 10 μΑ, corresponding to the Low Resistance State (LRS), which yields an ON/OFF ratio of >10^4^ for our device. The abrupt switching is indicative of filamentary switching, possibly induced by metallic Ag cations and/or iodine vacancies. When the external voltage amplitude becomes lower than a threshold, the device spontaneously relaxes to the HRS as the current reduces from 0.1 mA/10 μΑ back to the few nA range, indicating that the device resistance around zero bias is HRS, independent of the scanning direction (i.e., from low voltage amplitude to high or vice versa). This behavior is consistent with threshold RS, as the device sets after a threshold voltage and then relaxes back to the HRS. We also observe, in the case of compliance currents of 100 μA and 10 μΑ, a distinct difference in the switching voltage. To further study this behavior, we plotted the threshold switching voltage for both compliance currents of 10 μΑ and 100 μΑ. The box plot in Figure 3c shows a difference of approximately 1 V between the 2 cases. In more detail, the average threshold voltage for 100 μA was (3.16 ± 0.29) V, while for 10 μA it was (4.15 ± 0.33) V. This difference in the switching voltage is expected, as the formation process for the case of 100 μA is stronger, possibly resulting in the formation of a relatively stronger filament compared to the 10 μA case; hence, the threshold switching voltage required for the SET process is reduced. A performance comparison between our device and current state-of-the-art lead-free compounds can be found in Table S1.

To elucidate the reproducibility of the prepared devices, data from single cells belonging to 3 different batches of experiments are shown in Figure S3. No considerable difference was observed in the current–voltage characteristics, indicating a small variation from batch to batch, thereby confirming the reproducibility of our devices. The lifetime stability of the samples was also tested by performing aging tests. Specifically, the current–voltage characteristics of fresh samples and those aged for 2 weeks of storage in the glovebox are presented in Figure S4. The small variation reported is within the stochastic behavior of filament formation upon bias poling (see variations in Figure 3 after 10 consecutive I–V curves); therefore, no significant effect on device lifetime is reported after 2 weeks.

**Figure 4:**
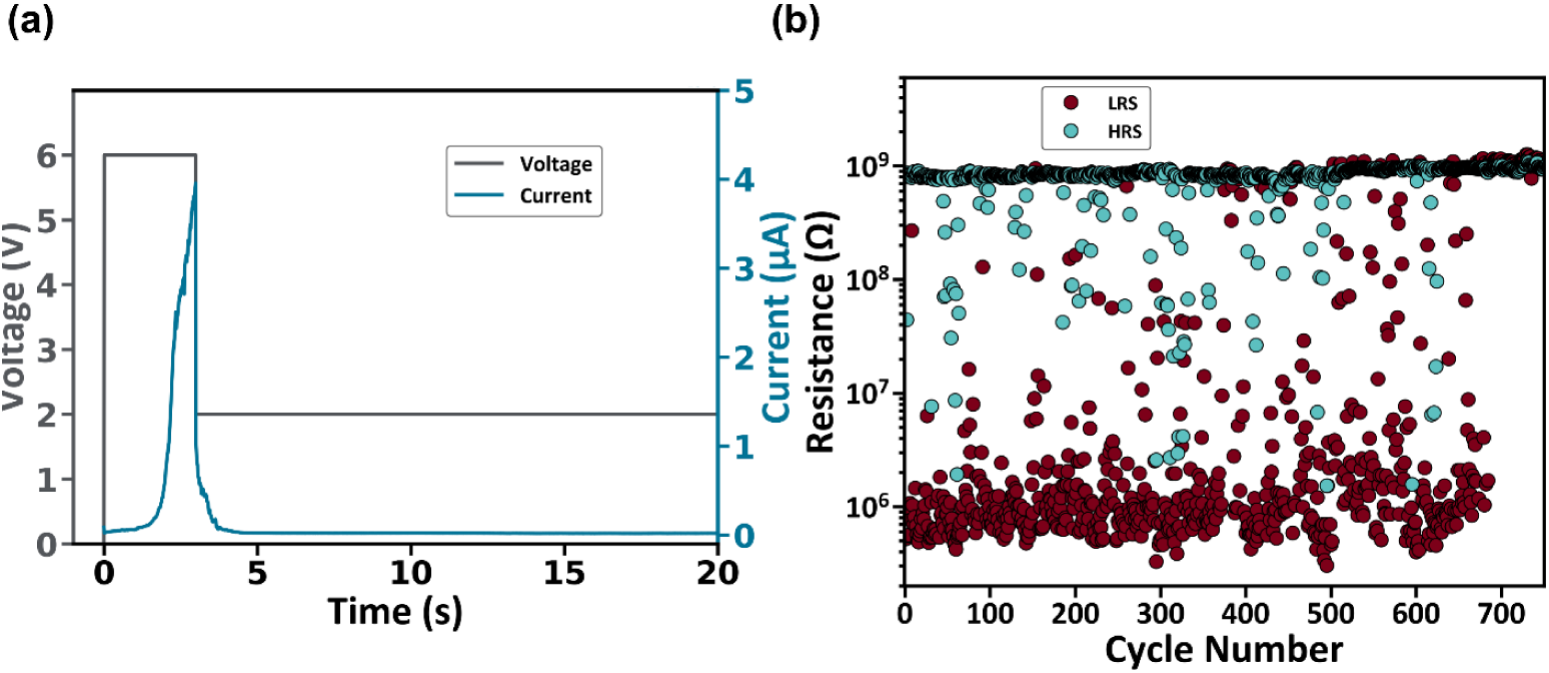
(a) Electric pulse measurement to confirm the volatile nature of the CBI device. A 6 V pulsed is used to set the device at LRS, and then a read pulse at 2 V measures the current decay back to the HRS. (b) Cycling endurance of the CBI device.

We continued our investigation by using electrical pulses to provide more insight into the device’s operational mechanism. Since we observed threshold switching during the steady-state I–V measurements, the volatile nature of the device should also be confirmed using voltage pulses. According to Figure 4a, an electrical pulse with an amplitude of 6 V was applied to the device for a duration of 3 s, which was sufficient to set the device to the LRS. Then, a constant read pulse was applied with an amplitude of 2 V for several seconds to record the current sample’s response. Interestingly, during the SET voltage pulse, the current gradually increased to ≈4 μA, and when the reading pulse started, the current rapidly reduced to 10 nA, which is a value similar to the steady-state measurements, confirming the volatile behavior of the device. As a next step, the cycling endurance of the device was also evaluated by implementing a write–read–erase–read pulse sequence. The write pulse had an amplitude of 5 V and a width of 1 s, while the erase pulse had an amplitude of −1 V and a width of 1 s. Finally, a read pulse of 2 V for 100 ms was used to extract resistance values for both states. A typical endurance plot is shown in Figure 4b. The LRS values are on the order of 1 MΩ, while the HRS values are on the order of 1 GΩ; hence, the ON/OFF ratio is ≈10^3^. This ratio was maintained for approximately 650 cycles, after which the LRS abruptly transitioned to the HRS, leading to a spontaneous device relaxation to the initial HRS.

**Figure 5:**
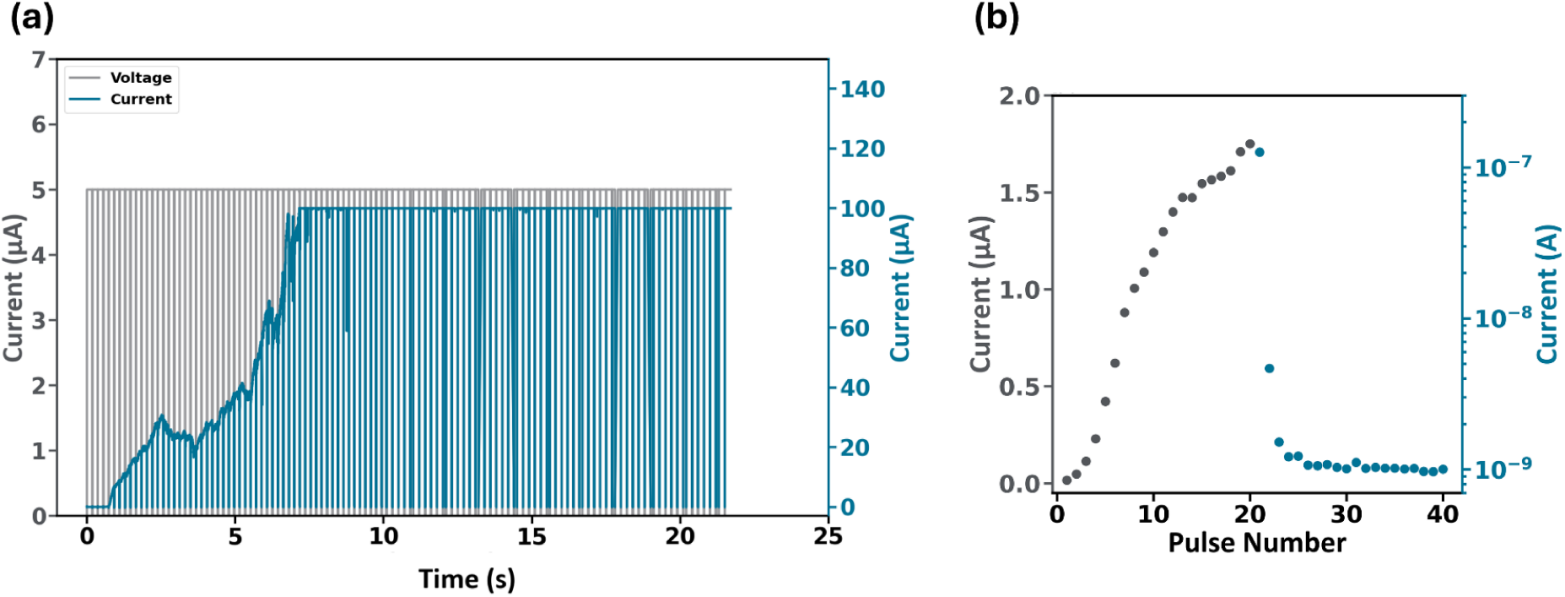
(a) Observation of threshold firing-like behavior under 100 identical pulses (b) Long-term potentiation and relaxation of the CBI device.

We now proceed to further study the threshold behavior of the lead-free perovskite memristive system. A sequence of 100 identical pulses, each with an amplitude of 5 V and a width of 180 ms, was applied. As seen in Figure 5a, during the initial pulses, the measured current is on the order of a few nA, and then it abruptly increases to the μA level, followed by a further increase until it reaches the compliance value set at 100 μA. These findings are consistent with the threshold behavior of our device, supporting a neuron-like behavior.^62^

Consequently, we demonstrate long-term potentiation (LTP) followed by relaxation, as shown in Figure 5b. Two trains of identical pulses were applied as follows: 20 identical pulses of 2 V in amplitude and having a duration of 50 ms in width to induce LTP, equivalent to the observed gradual current increase, and 20 identical pulses of −500 mV in amplitude and 50 ms in width to induce the current decay, which is consistent with the volatile dynamics in our device. Additional data for different pulse amplitudes and widths are included in Figure S5, indicating their on LTP/STP dynamics.

**Figure 6:**
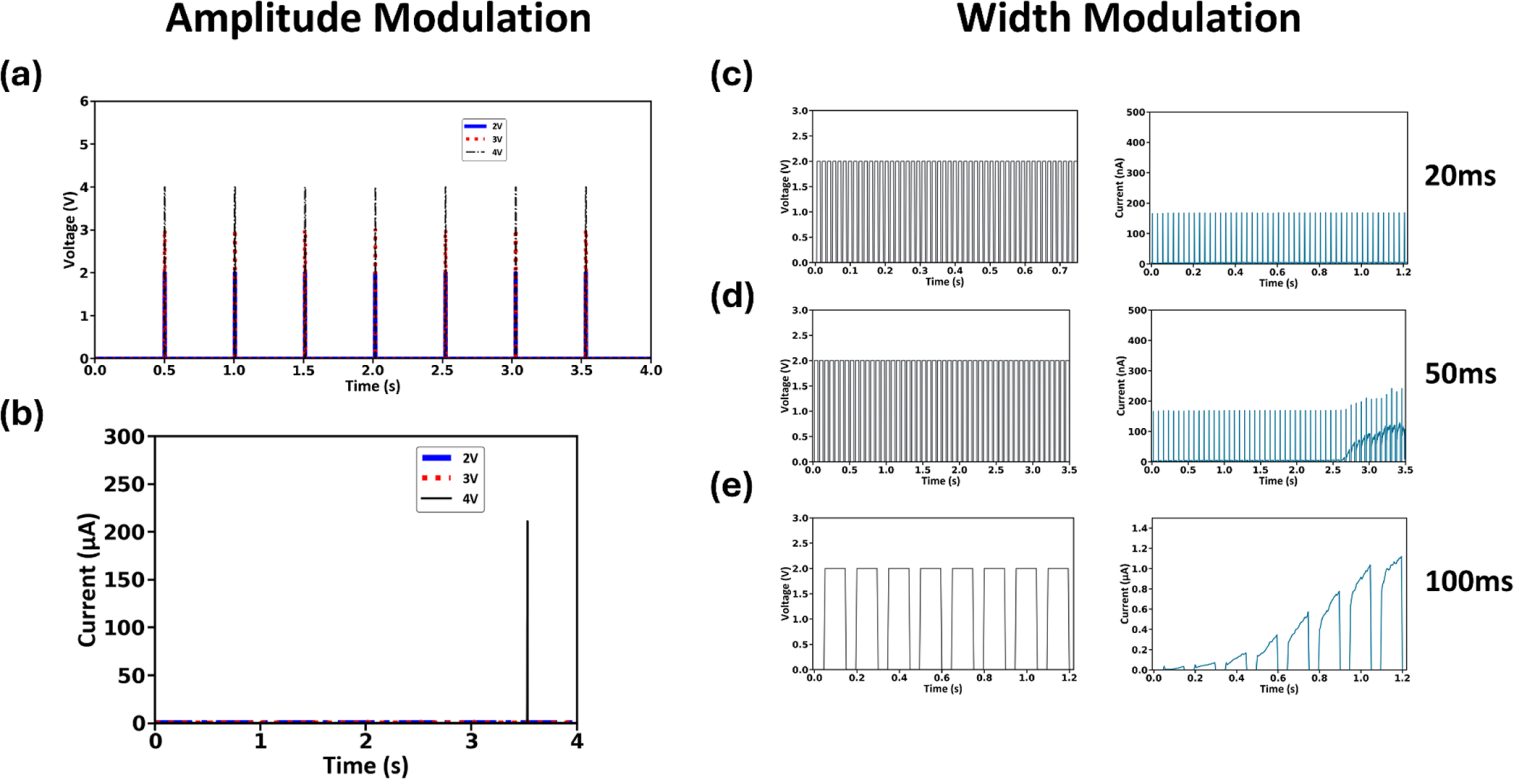
Observation of current spiking in the device after a threshold similar to that for neuron spiking. The measurement was repeated for identical pulses of 2 V, 3 V, and 4 V amplitude as shown in (a), where the corresponding current response is shown in (b). The threshold behavior was confirmed by modulating the pulse width at (c) 20 ms, (d) 50 ms, and (e) 100 ms, while keeping the amplitude fixed at 2 V.

We proceeded to test the function of our device as a single, simple electronic component for neuron emulation. A series of seven identical pulses were applied, having varying amplitudes of 2 V, 3 V, and 4 V (Figure 6a; different colors correspond to different amplitudes). Each pulse had a duration of 5 ms. During all measurements, the compliance current was set at 1 mA, and the time interval between each pulse was fixed at 20 ms. In the case of pulses with amplitudes of 2 V and 3 V, the current response of the device was on the order of ns (Figure 6b). When the amplitude was increased to 4 V, a current spike of approximately 200 μA was observed. This indicates a threshold behavior occurring to activate the device, a behavior that resembles neuron-like characteristics, emulating a threshold, firing-like process.

We next investigate the response of the device when varying the pulse width, while the amplitude is fixed at 2 V. In the first case, shown in Figure 6c, no current spiking is observed when the pulse width is set to 20 ms. When the width is increased to 50 ms (Figure 6d), a slight current increase is observed after approximately 2.5 s. Finally, further increasing the width to 100 ms (Figure 6e) leads to an abrupt current enhancement, which requires a smaller number of pulses compared to the 50 ms case. This confirms the observation of threshold characteristics in our device by modulating either the amplitude or the pulse width.

**Figure 7:**
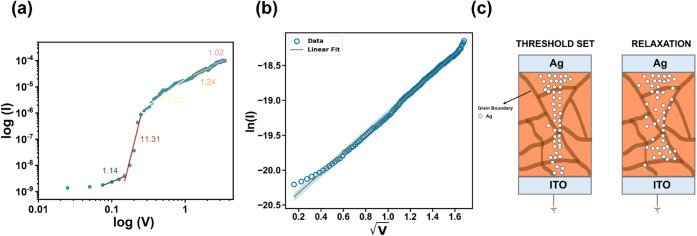
(a) Conduction mechanism analysis based on the I–V characteristics at the initial stages of LRS and during relaxation back to HRS. (b) I–V analysis for the conduction mechanism in the HRS. Linear relationship between ln(I) and  indicates Schottky emission. (c) Simplified schematic illustration of the mechanism governing the volatile, diffusive dynamics of the RS device.

As a final step, we focus on providing more insight into the operational mechanism of our volatile RS system (Figure 7). The abrupt threshold switching observed is a strong indicator of filamentary switching that most likely originates from Ag cation migration through an electrochemical process. The thickness of the perovskite layer (∼900 nm) may also contribute to the presence of transient, weak Ag filaments that decay upon electric field removal, leading to device restoration back to the initial HRS. We plot in Figure 7 the I–V characteristics for both LRS and HRS to confirm the mechanism of conduction. At the LRS, ohmic conduction is observed as current varies linearly (slope of 1.02) with voltage, which is indicative of filamentary switching. Conversely, as the device begins to relax back to the initial HRS, the conduction mechanism changes to a charge trapping–detrapping process, as the three main regions of conduction are observed:^50,63^ ohmic conduction with a slope of 1.14, trap filling with a slope of 11.31, space-charge-limited current (SCLC) region with a slope of 2.02 and, finally, ohmic-like conduction with a slope of 1.24 (Figure 7a). At the HRS (Figure 7b) the square root of the voltage varies linearly with ln(I), which indicates Schottky emission. A plausible explanation for the observed volatile behavior is the trapping of Ag metal cations, which causes the disappearance of the conductive filament and the relaxation of the device to HRS. A simplified picture of the filament formation and rupture processes is depicted in Figure 7c.

## Conclusions

In this study, we demonstrate the fabrication of an inorganic lead-free perovskite memristive device with threshold switching behavior using solution-based manufacturing. The resulting device exhibited an ON/OFF ratio of ∼10^4^ while operating within the 0 V–5 V range and showed reproducible switching characteristics across different samples from the same batch or different batches tested. Extended pulsed characterization protocols confirmed the volatile nature of the system, with a volatile cycling endurance of approximately 650 cycles demonstrated. Furthermore, linear long- term potentiation protocols under threshold switching, accompanied by abrupt resistance suppression under depression protocols, were observed . The volatile nature of memristive switching enabled the implementation of random current spiking activation, similar to neuron spiking protocols, thus opening the path for neuronal emulation using a simple, single-device component with a small footprint compared to implementations based on silicon or other oxide systems that require more complex circuits with multiple passive and active components and more intricate fabrication procedures.

## Supporting Information Available

Comparison table with state-of-the-art devices, Tauc plot from UV–vis. Data to obtain the perovskite bandgap, profilometer measurements for thickness estimation of the perovskite film, current–voltage measurements from three different batches, current–voltage characteristics after 2 weeks of storage in glovebox, additional LTP-relaxation data (PDF)
