# Supplementary material for: Threshold Resistive Switching in Inorganic Lead-Free Cesium–Bismuth Iodide Perovskite for Neuron Emulation
Source: ACS Appl Electron Mater. 2025 Apr 4;7(8):3610–9. doi: 10.1021/acsaelm.5c00516 (PMC12020440; doi:10.1021/acsaelm.5c00516)
Supplement: Supplementary file 1 — el5c00516_si_001.pdf [file el5c00516_si_001.pdf]

Supporting Information

Threshold Resistive Switching in Inorganic Lead-Free Cesium-Bismuth Iodide Perovskite for Neuron Emulation

Michalis Loizos[1, ‡], Konstantinos Chatzimanolis[1, ‡], Katerina Anagnostou[1], Konstantinos Rogdakis[1,2*], and Emmanuel Kymakis[1,2*]

‡ These authors contributed equally

[1]Department of Electrical & Computer Engineering, Hellenic Mediterranean University (HMU), Heraklion 71410, Crete, Greece

[2]Institute of Emerging Technologies, University Research and Innovation Center, HMU, Heraklion 71410, Crete, Greece

Corresponding Author

***Konstantinos Rogdakis** Email: krogdakis@hmu.gr
***Emmanuel Kymakis** Email: kymakis@hmu.gr

Table S1 Performance summary of lead-based and lead-free perovskite threshold switching devices

	Compound	Deposition Method	Operation Mode	ON/OFF Ratio	Threshold Voltage V_{TH} (V)	Year	Ref.
1	Single crystal $Cs_2AgBiBr_6$	Pressure-assisted Crystallization	Non-Volatile to Volatile	10^7	≈ 1	2022	1

2	$Cs_3Sb_2Br_9$	Direct dripping & Crystallization	Non-Volatile to Volatile	10^6	≈ ± 2	2020	[2]
3	$Cs_3Cu_2Cl_5$	Spin-coating	Non-Volatile to Volatile	$\approx 10^2$	0.6	2022	[3]
4	$FAPbI_3$	Spin-coating	Volatile	10^7	0.075	2024	[4]
5	$Cs_2AgBiBr_6$	Spin-coating	Non-Volatile to Volatile	$> 10^2$	± 0.6	2023	[5]
6	$CsCu_2I_3$	Spin-coating	Volatile	10^4	0.54	2022	[6]
7	$MAPbI_3$	Spin-coating	Volatile	10^5	0.4	2020	[7]
8	$MAPbBr_3$	Spin-coating	Volatile	10^2	0.65	2024	[8]
9	$CsPbBr_3$ Nanocrystals	Spin-coating	Non-Volatile to Volatile	$> 10^3$	0.5	2022	[9]
10	$C_{12}H_{36}BiI_6N_3$	Spin-coating	Volatile	2.2×10^3	0.23	2024	[10]
11	$MAPbI_3$	Spin-coating	Volatile	10^4	0.8	2023	[11]
12	$RbPbI_3$	Spin-coating	Non-Volatile to Volatile	10^3(Volatile)	2.5	2023	[12]

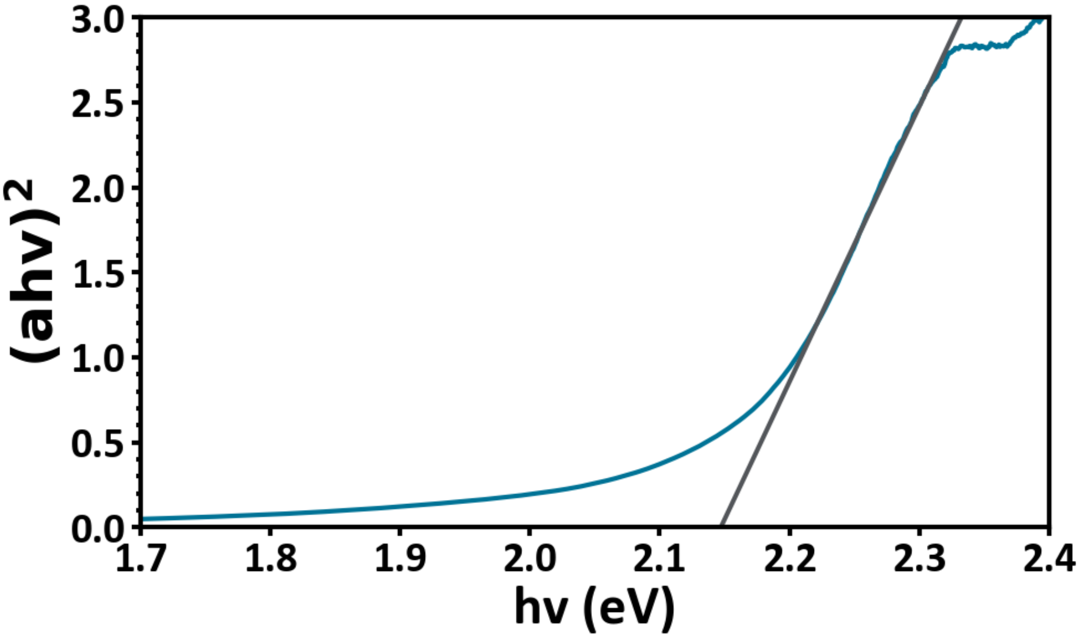

Figure S1 Bandgap estimation of the CBI perovskite through the Tauc plot method.

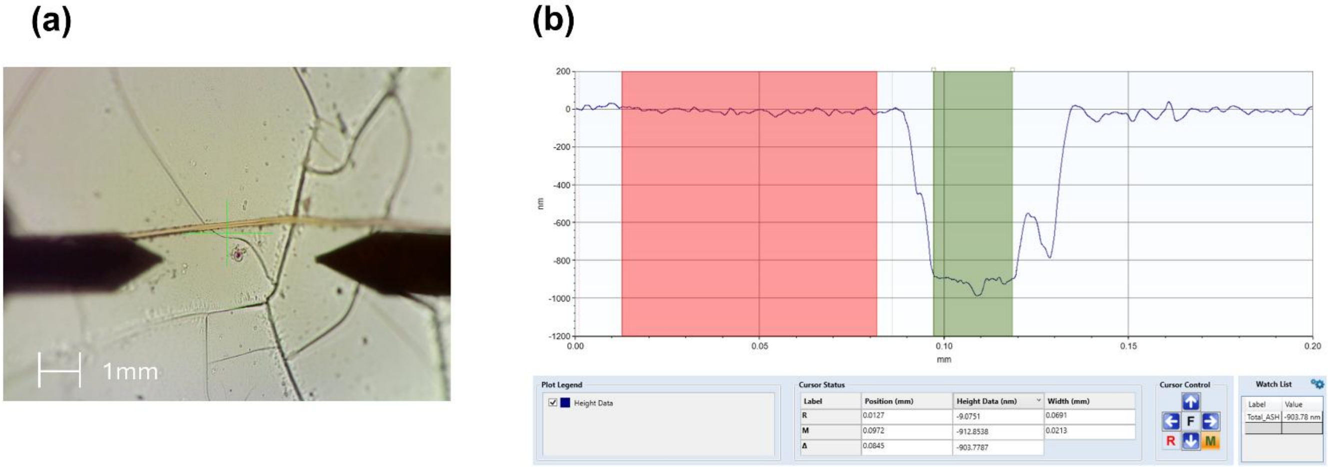

Figure S2 (a) Optical image of the CBI perovskite surface. **(b)** Thickness estimation of the Lead-free perovskite film through profilometer.

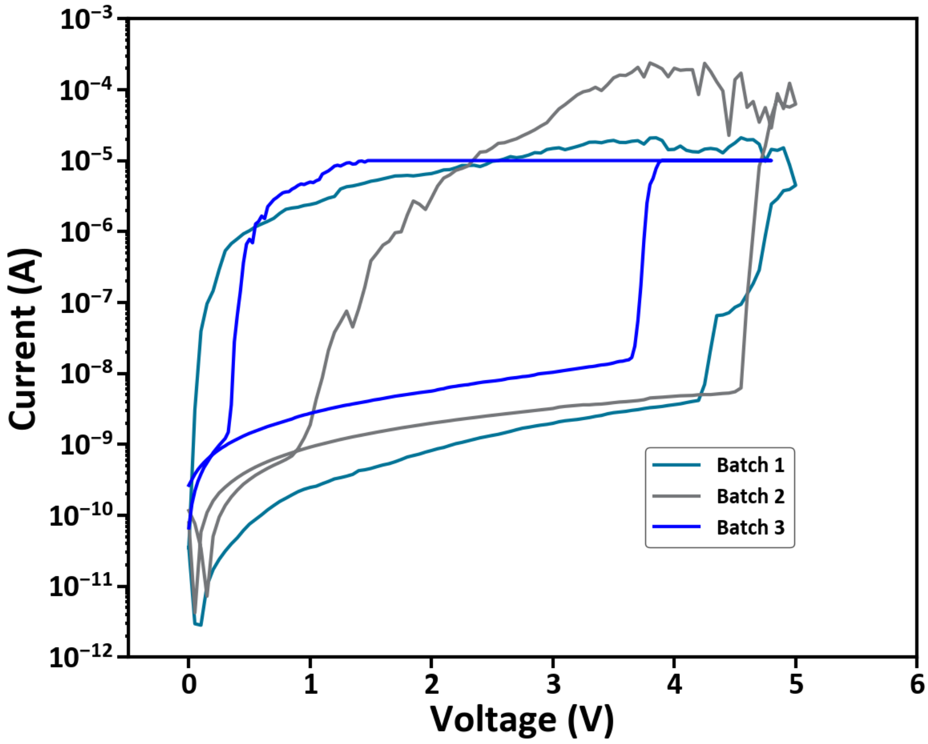

Figure S3 Current-Voltage characteristics of CBI-based devices for single cells belonging in 3 different batches.

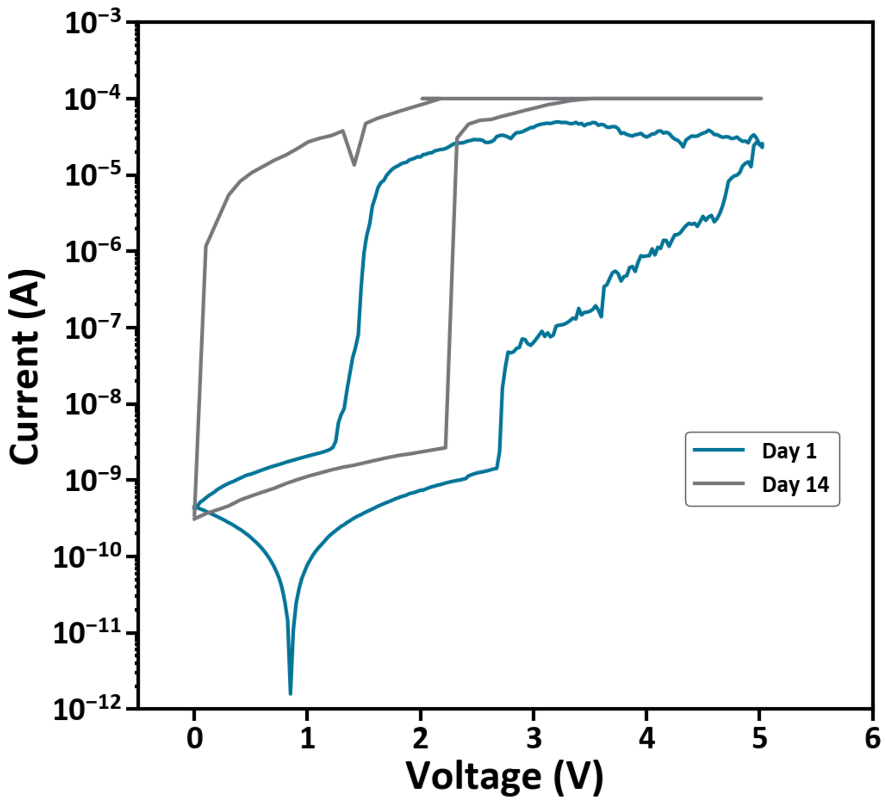

Figure S4 Current-Voltage characteristics of the CBI devices within 2 weeks of storage in a nitrogen-filled glovebox.

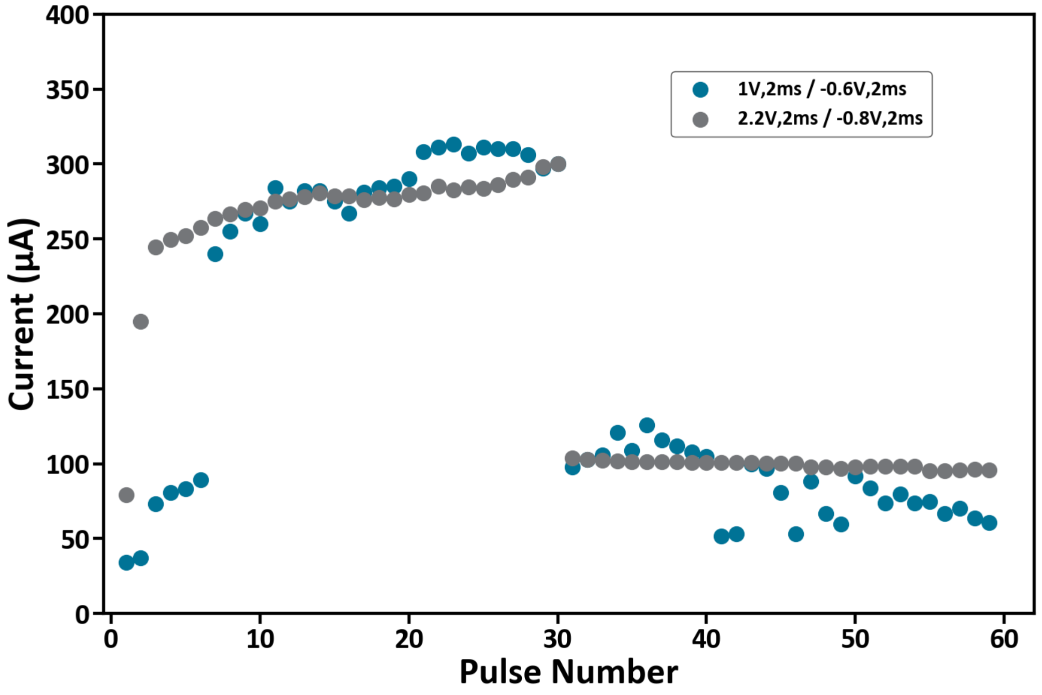

Figure S5 Additional Potentiation/Relaxation data for different pulse amplitude and width.

References

(1) You, Q.; Huang, F.; Fang, F.; Zhu, J.; Zheng, Y.; Fang, S.; Zhou, B.; Li, H.; Han, C.; Shi, Y. Controllable Volatile-to-Nonvolatile Memristive Switching in Single-Crystal Lead-Free Double Perovskite with Ultralow Switching Electric Field. *Sci. China Mater.* **2023**, *66* (1), 241–248. https://doi.org/10.1007/s40843-022-2113-y.

(2) Mao, J.-Y.; Zheng, Z.; Xiong, Z.-Y.; Huang, P.; Ding, G.-L.; Wang, R.; Wang, Z.-P.; Yang, J.-Q.; Zhou, Y.; Zhai, T.; Han, S.-T. Lead-Free Monocrystalline Perovskite Resistive Switching Device for Temporal Information Processing. *Nano Energy* **2020**, *71*, 104616. https://doi.org/10.1016/j.nanoen.2020.104616.

(3) He, N.; Ye, F.; Liu, J.; Sun, T.; Wang, X.; Hou, W.; Shao, W.; Wan, X.; Tong, Y.; Xu, F.; Sheng, Y. Multifunctional Ag–In–Zn–S/$Cs_3Cu_2Cl_5$-Based Memristors with Coexistence of Non-Volatile Memory and Volatile Threshold Switching Behaviors for Neuroinspired Computing. *Advanced Electronic Materials* **2023**, *9*(3), 2201038. https://doi.org/10.1002/aelm.202201038.

(4) Li, Y.; Li, J.; Ni, J.; Zhang, J.; Cai, H. Low-Power Perovskite-Based Threshold Switching Memristor for Artificial Nociceptor. *Journal of Alloys and Compounds* **2024**, *1000*, 175121. https://doi.org/10.1016/j.jallcom.2024.175121.

(5) Huang, M.; Hou, M.; Xing, H.; Tu, J.; Jia, S. Transient Memristive Device Based on Lead-Free Double Perovskite for Secured Data Storage and Artificial Learning Systems. *Ceramics International* **2023**, *49*(7), 10365–10374. https://doi.org/10.1016/j.ceramint.2022.11.217.

(6) Huang, F.; Ge, S.; Wei, R.; He, J.; Ma, X.; Tao, J.; Lu, Q.; Mo, X.; Wang, C.; Pan, C. Flexible Threshold Switching Based on $CsCu_2I_3$ with Low Threshold Voltage and High Air Stability. *ACS Appl. Mater. Interfaces* **2022**, *14*(38), 43474–43481. https://doi.org/10.1021/acsami.2c09904.

(7) Yang, J.-Q.; Wang, R.; Wang, Z.-P.; Ma, Q.-Y.; Mao, J.-Y.; Ren, Y.; Yang, X.; Zhou, Y.; Han, S.-T. Leaky Integrate-and-Fire Neurons Based on Perovskite Memristor for Spiking Neural Networks. *Nano Energy* **2020**, *74*, 104828. https://doi.org/10.1016/j.nanoen.2020.104828.

(8) Gonzales, C.; Bou, A.; Guerrero, A.; Bisquert, J. Capacitive and Inductive Characteristics of Volatile Perovskite Resistive Switching Devices with Analog Memory. *J. Phys. Chem. Lett.* **2024**, *15*(25), 6496–6503. https://doi.org/10.1021/acs.jpclett.4c00945.

(9) John, R. A.; Demirağ, Y.; Shynkarenko, Y.; Berezovska, Y.; Ohannessian, N.; Payvand, M.; Zeng, P.; Bodnarchuk, M. I.; Krumeich, F.; Kara, G.; Shorubalko, I.; Nair, M. V.; Cooke, G. A.; Lippert, T.; Indiveri, G.; Kovalenko, M. V. Reconfigurable Halide Perovskite Nanocrystal Memristors for Neuromorphic Computing. *Nat Commun* **2022**, *13*(1), 2074. https://doi.org/10.1038/s41467-022-29727-1.

(10) Zawal, P.; Abdi, G.; Gryl, M.; Das, D.; Sławek, A.; Gerouville, E. A.; Marciszko-Wiąckowska, M.; Marzec, M.; Hess, G.; Georgiadou, D. G.; Szaciłowski, K. Leaky Integrate-and-Fire Model and Short-Term Synaptic Plasticity Emulated in a Novel

Bismuth-Based Diffusive Memristor. *Advanced Electronic Materials* **2024**, *10* (7), 2300865. https://doi.org/10.1002/aelm.202300865.

(11) Patil, H.; Kim, H.; Kadam, K. D.; Rehman, S.; Patil, S. A.; Aziz, J.; Dongale, T. D.; Ali Sheikh, Z.; Khalid Rahmani, M.; Khan, M. F.; Kim, D. Flexible Organic–Inorganic Halide Perovskite-Based Diffusive Memristor for Artificial Nociceptors. *ACS Appl. Mater. Interfaces* **2023**, *15*(10), 13238–13248. https://doi.org/10.1021/acsami.2c16481.

(12) Das, U.; Nyayban, A.; Paul, B.; Barman, A.; Sarkar, P.; Roy, A. Compliance Current-Dependent Dual-Functional Bipolar and Threshold Resistive Switching in All-Inorganic Rubidium Lead-Bromide Perovskite-Based Flexible Device. *ACS Appl. Electron. Mater.* **2020**, *2*(5), 1343–1351. https://doi.org/10.1021/acsaelm.0c00130.
